# CNOT4-Mediated Ubiquitination of Influenza A Virus Nucleoprotein Promotes Viral RNA Replication

**DOI:** 10.1128/mBio.00597-17

**Published:** 2017-05-23

**Authors:** Yu-Chen Lin, King-Song Jeng, Michael M. C. Lai

**Affiliations:** a Institute of Molecular Biology, Academia Sinica, Taipei, Taiwan; b National RNAi Core Facility, Academia Sinica, Taipei, Taiwan; c Research Center for Emerging Viruses, China Medical University Hospital, China Medical University, Taichung, Taiwan; Columbia University

**Keywords:** CNOT4, IAV, NP, RNA replication, influenza A virus, nucleoprotein, ubiquitination

## Abstract

Influenza A virus (IAV) RNA segments are individually packaged with viral nucleoprotein (NP) and RNA polymerases to form a viral ribonucleoprotein (vRNP) complex. We previously reported that NP is a monoubiquitinated protein which can be deubiquitinated by a cellular ubiquitin protease, USP11. In this study, we identified an E3 ubiquitin ligase, CNOT4 (Ccr4-Not transcription complex subunit 4), which can ubiquitinate NP. We found that the levels of viral RNA, protein, viral particles, and RNA polymerase activity in CNOT4 knockdown cells were lower than those in the control cells upon IAV infection. Conversely, overexpression of CNOT4 rescued viral RNP activity. In addition, CNOT4 interacted with the NP in the cell. An *in vitro* ubiquitination assay also showed that NP could be ubiquitinated by *in vitro*-translated CNOT4, but ubiquitination did not affect the protein stability of NP. Significantly, CNOT4 increased NP ubiquitination, whereas USP11 decreased it. Mass spectrometry analysis of ubiquitinated NP revealed multiple ubiquitination sites on the various lysine residues of NP. Three of these, K184, K227, and K273, are located on the RNA-binding groove of NP. Mutations of these sites to arginine reduced viral RNA replication. These results indicate that CNOT4 is a ubiquitin ligase of NP, and ubiquitination of NP plays a positive role in viral RNA replication.

## IMPORTANCE

Influenza virus, particularly influenza A virus, causes severe and frequent outbreaks among human and avian species. Finding potential target sites for antiviral agents is of utmost importance from the public health point of view. We previously found that viral nucleoprotein (NP) is ubiquitinated, and ubiquitination enhances viral RNA replication. In this study, we found a cellular ubiquitin ligase, CNOT4, capable of ubiquitinating NP. The ubiquitination sites are scattered on the surface of the NP molecule, which is critical for RNA replication. CNOT4 and a ubiquitin protease, USP11, together regulate the extent of NP ubiquitination and thereby the efficiency of RNA replication. This study thus identifies a potential antiviral target site and reveals a novel posttranslational mechanism for regulating viral replication. This represents a novel finding in the literature of influenza virus research.

## INTRODUCTION

Influenza A virus (IAV) is a major pathogen that causes human respiratory diseases and leads to significant morbidity and mortality every year (1). Like all other viruses, IAV replication relies on many proteins or enzymes from the virus itself and the host cell (2). It is important to understand the mechanism of virus-host cell interaction to identify new therapeutic targets and develop antiviral drugs (3). IAV is a member of the *Orthomyxoviridae* family, whose genome consists of eight single-stranded, negative-sense RNA segments that encode 14 proteins (4). Replication and transcription of viral RNA (vRNA) requires the viral ribonucleoprotein (vRNP) complex, which consists of viral nucleoprotein (NP) and three viral RNA polymerase subunits (PB1, PB2, and PA) (5). The viral RNP complex synthesizes three types of viral RNA (vRNA, mRNA, and cRNA). Because of the small size of the viral genome, it is natural that some components of the replication machinery are provided by the host (6). Therefore, cellular factors that associate with the vRNP likely play important roles in the viral life cycle (7). Recently, several independent genome-wide RNA interference (RNAi) screens have identified over a thousand human genes that could participate in IAV infection and replication (8–12).

The posttranslational modification (PTM) of cellular factors leads to complex signals, including short- and long-term regulation of viral infection (13). The ubiquitination pathway is one of the PTM machineries; it regulates a cellular signal that labels proteins in a highly controlled manner by changing the stability, localization, or activity of the target protein (14). Several recent studies on IAV-associated ubiquitin modifications showed that ubiquitination of viral proteins often leads to degradation of the proteins by proteasomes. For example, ubiquitin ligase TRIM22 catalyzes NP polyubiquitination (15), while TRIM32 targets the PB1 polymerase for ubiquitination (16); both ubiquitination events result in protein degradation. Thus, in these cases, ubiquitination acts as a host defense response to IAV infection by degrading the essential viral or cellular proteins. Moreover, IAV NS1 protein inhibits TRIM25-mediated RIG-I CARD ubiquitination, thereby suppressing RIG-I signal transduction and inhibiting the host interferon (IFN) system (17, 18). In this case, ubiquitin serves to disrupt the cellular defense machineries against viral infections.

Still another type of ubiquitination in IAV is represented by M1 protein ubiquitination. We found that an E3 ubiquitin ligase, Itch, is incorporated into the endosome during virus entry; ubiquitination of viral M1 protein by Itch triggers vRNP egress into the cytosol by an unknown mechanism and also the transport of vRNP into the nucleus, where vRNP replicates (19). Free ubiquitin in the virion has also been implicated in this process (20). Finally, we have reported yet another ubiquitination event of IAV proteins, namely, the ubiquitination/deubiquitination of NP protein. We showed that a deubiquitinase, USP11, can remove ubiquitin from NP and suppress viral RNA replication (21), implying that ubiquitinated NP may act as a positive factor for IAV replication. This is in contrast to the reported TRIM22-medicated NP ubiquitination, which causes NP degradation (15). Based on these considerations, we attempted to identify the ubiquitin machinery that facilitates IAV RNA replication.

Conjugation of ubiquitin to a target protein is regulated by the sequential activity of ubiquitin-activating (E1), ubiquitin-conjugating (E2), and ubiquitin-ligating (E3) enzymes. It typically results in the addition of a ubiquitin moiety either to the ε-amino group of a lysine residue or to the extreme amino terminus of a polypeptide (22). Specific E3 enzymes are responsible for the selectivity of ubiquitin-protein ligation, and the pairing of specific enzymes with cognate substrates allows for exquisite specificity in regulating substrate modification (23).

In this study, we used a small-scale RNAi screen to search for E3 ligases that may facilitate IAV replication. We found that Ccr4-Not transcription complex subunit 4 (CNOT4) acts as a positive regulator for IAV replication in early steps of the viral replication cycle. The Ccr4-Not complex consists of nine core subunits in *Saccharomyces cerevisiae*, and homologs of all these subunits exist in most of the other eukaryotes (24). CNOT4 has ubiquitin ligase activity which regulates mRNA transcription, metabolism, and proteasome assembly in cells (25). We demonstrate here that CNOT4 is a ubiquitin ligase capable of ubiquitinating NP, resulting in the enhancement of viral RNP activity. The removal of ubiquitin by knockdown of CNOT4 or treatment with USP11 suppressed NP ubiquitination and viral RNA replication. The ubiquitin ligase CNOT4 and ubiquitin protease USP11 serve as competing enzymes in the regulation of NP ubiquitination.

## RESULTS

### Search for E3 ligase genes from pooled RNAi screen.

**FIG 1:**
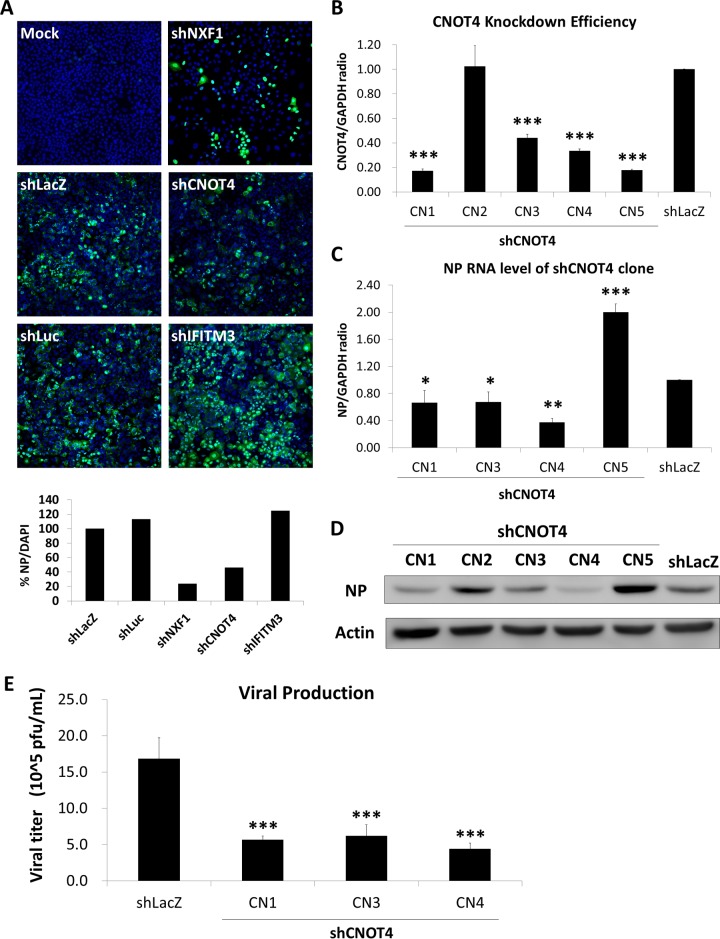
Knockdown of CNOT4 inhibits IAV replication. (A) Immunofluorescence analysis of secondary RNAi screening. A549 cells were transduced with various shRNA-harboring lentiviruses that targeted the different genes identified in the primary pooled shRNA library screen (19). The transduced cells were then infected with WSN/33 virus at an MOI of 1. At 6 h p.i., the cells were subjected to immunofluorescence staining using NP antibody and DAPI. The intensity of NP-DAPI was used as an indicator and normalized to control shLacZ staining intensity to estimate the percentage of cells infected with IAV. (B and C) CNOT4 knockdown efficiency (B) and relative viral NP RNA levels (C) were determined by qRT-PCR, using RNA from the various CNOT4 knockdown A549 cells (shRNA clones CN1 to CN5) at 6 h p.i. (means ± SD, *n* = 4). *, *P* < 0.05; **, *P* < 0.01; ***, *P* < 0.001, based on one-way ANOVA with Dunnett’s multiple-comparison test. (D) Viral proteins were detected by immunoblotting at 6 h p.i. with an anti-NP antibody. Actin was used as an internal control. (E) Virus titers from progeny virus released into the medium were determined at 24 h p.i. via plaque assay on MDCK cells (means ± SD, *n* = 3). ***, *P* < 0.001, based on one-way ANOVA with Dunnett’s multiple-comparison test.

Previously, we performed a pooled genome-wide RNAi library screen to identify candidate cellular genes that potentially affect IAV replication (19). Among the candidate genes identified, 11 of them were E3 ligases. For each candidate gene, we then used four to five different shRNA clones to knock down their gene expression in A549 cells. The cells were then infected with influenza A virus strain WSN and subjected to immunofluorescence analysis of viral NP at 6 h postinfection (p.i.) (Fig. 1A). We used the intensity of NP stained with 4′,6-diamidino-2-phenylindole (DAPI) as an indicator, and results were normalized to results with the control shLacZ to estimate the percentages of infected cells. NXF1 (nuclear RNA export factor 1), which has been reported to affect IAV replication (26), was used as a positive control. Another control was IFITM3 (interferon-inducible transmembrane protein 3), which is required for antiviral responses involving IFN (27). Among all the candidate E3 ligase genes, only CNOT4 gave consistent results (data not shown). Knockdown of CNOT4 decreased the viral NP level by 60%, suggesting that CNOT4 is required for the replication of IAV. We proceeded to study the possible role of CNOT4 E3 ligase in IAV replication.

### Knockdown of CNOT4 inhibits IAV replication.

We next examined the step in the virus life cycle affected by CNOT4. A549 cells were infected with five different CNOT4 shRNA-expressing lentiviruses (clones CN1 to CN5) to induce knockdown of CNOT4 and then infected the cells with strain WSN virus. The total intracellular RNA was harvested at 6 h p.i. for quantitative reverse transcription-PCR (qRT-PCR) analysis of RNA and immunoblotting of viral proteins (Fig. 1B to D). The shRNA clones CN1, CN3, and CN4 showed CNOT4 knockdown efficiencies of 60 to 80% (Fig. 1B); correspondingly, viral NP RNA was reduced to about 40 to 60% (Fig. 1C), and the NP protein level was reduced about 30 to 70% (Fig. 1D). CN2 did not suppress CNOT4 and was excluded from further studies (Fig. 1B and D). It is noteworthy that CN2 expressed normal amounts of viral protein (Fig. 1D). CN5 reduced CNOT4 expression but did not suppress NP expression (Fig. 1B to D). This clone might have off-target effects and was not studied further. Consistent with these RNA and protein expression profiles, CN1, -3, and -4 also produced lower virus titers than the control cells (Fig. 1E). All these results indicated that CNOT4 depletion suppresses IAV replication. CN1 and CN3 were chosen for further study, because these two clones showed the most consistent phenotype and used different mechanisms for CNOT4 suppression. From the RNAi Core Facility, it is known that the shRNA of CN1 targets the 3′-untranslated region (UTR) of the CNOT4 mRNA, whereas CN3 targets its coding region, thus giving independent verification of the experimental data for CNOT4.

### Viral RNP activity is correlated with the expression level of CNOT4.

**FIG 2:**
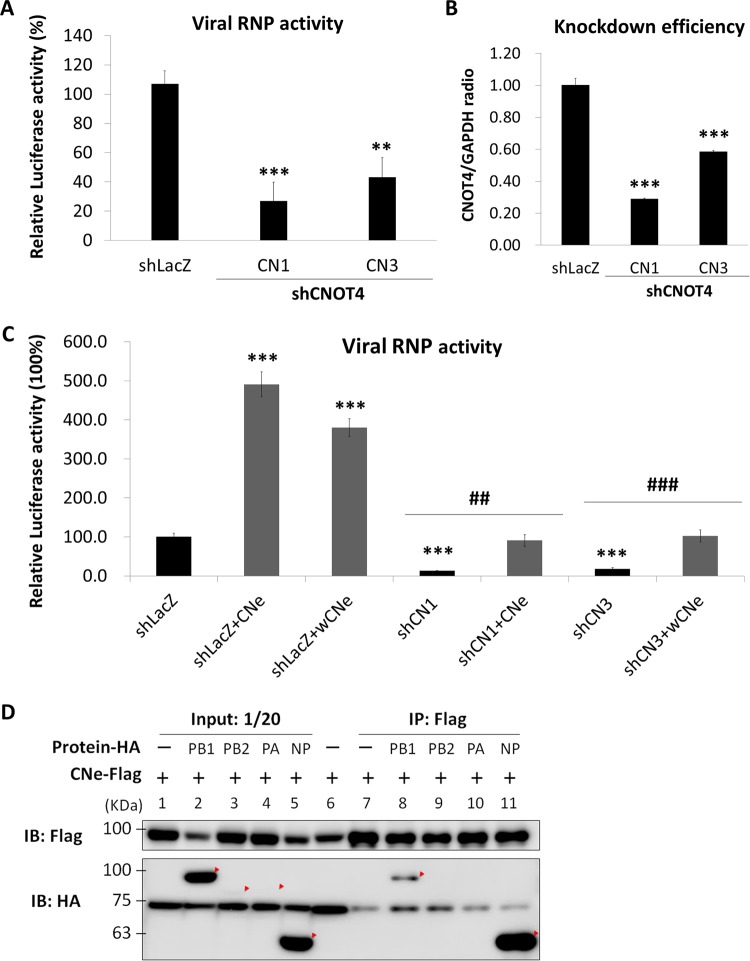
Viral RNP activity is correlated with the expression level of CNOT4. (A) Minireplicon assay results for RNP activity in CNOT4 knockdown cells. Two shCNOT4 clones, CN1 and CN3, and control shLacZ 293T cells were transfected with the plasmids for expression of viral PB1, PB2, PA, and NP and a reporter plasmid expressing the antisense luciferase gene as described elsewhere for the minireplicon assay (29). At 24 h posttransfection, luciferase activity was determined and normalized to the response of control shLacZ (means ± SD, *n* = 3). **, *P* < 0.01; ***, *P* < 0.001, by one-way ANOVA with Dunnett’s multiple-comparison test. (B) CNOT4 knockdown efficiency in 293T cells. (C) Overexpression of CNOT4 enhances viral RNP activity. CNOT4 knockdown (shRNA clones CN1 and CN3) or control cells (shLacZ) were transfected together with wild-type CNOT4 isoform e (CNe) or its wobble mutant (wCNe) and used for the minireplicon assay. Relative luciferase activities were measured as described for panel A (means ± SD, *n* = 3). ***, *P* < 0.001 (versus shLacZ); ##, *P* < 0.01; ###, *P* < 0.001 (versus shRNA clones CN1 or CN3), based on a one-way ANOVA with Tukey’s multiple-comparison test. (D) Interaction of CNOT4 with viral RNP components in an immunoprecipitation (IP) assay. 293T cells were cotransfected with Flag-tagged CNOT4 isoform e (CNe-Flag) and one of the HA-tagged viral proteins (PB1, PB2, PA, and NP). At 48 h posttransfection, cell lysates were precipitated using Flag-agarose. The proteins were visualized by Western blotting (IB) with anti-HA and anti-Flag antibodies.

Since CNOT4 has been reported to regulate transcription, decay of mRNA, translation, and protein degradation in the cell (28), we performed a minireplicon assay (29) to determine whether viral RNA transcription/replication was affected by CNOT4. The CNOT4 knockdown 293T cells were transfected with plasmids for the expression of viral RNP components (PB1, PB2, PA, and NP) and two indicator plasmids, pPolI-Luc as a reporter and *Renilla* luciferase as a transfection control. The results showed that knockdown of CNOT4 reduced viral RNP polymerase activity by about 60 to 70% (Fig. 2A), which correlated with the level of reduction of CNOT4 in these cells (Fig. 2B). To further confirm that the repression of viral RNP activity was caused by CNOT4 depletion, we overexpressed CNOT4 in the CNOT4 knockdown cells. Since the shRNA clone CN1 targets the 3′-UTR region of the CNOT4 mRNA, we used a plasmid expressing the wild-type CNOT4 isoform e (CNe) to determine whether the phenotype of the cells could be reversed. In contrast, the shRNA clone CN3 targets the CNOT4 coding region; we therefore used a plasmid expressing a wobble mutant of CNOT4 isoform e (wCNe), which had normal amino acids but a varied nucleotide sequence, to avoid it being targeted by the shRNA. Significantly, viral RNP activity was enhanced 4- to 5-fold in the control cells (shLacZ) when CNOT4 (either wild-type or the wobble mutant) was overexpressed. The wild-type or wobble mutant CNOT4 also rescued the RNP activity of the CNOT4 knockdown cells (shCN1 and shCN3) to nearly the level of the control cells (Fig. 2C, right). These results combined suggest strongly that CNOT4 can enhance the transcription/replication of IAV RNA.

To address the possible mechanism of RNP enhancement by CNOT4, we performed a coimmunoprecipitation assay to determine whether CNOT4 interacts with NP and/or other components of viral RNP, since NP is known to be ubiquitinated (15, 21). Cells were cotransfected with Flag-tagged CNOT4 and hemagglutinin (HA)-tagged NP, PB1, PB2, and PA. The cell lysates were immunoprecipitated with anti-Flag antibody in agarose. As shown in Fig. 2D, CNOT4 primarily coprecipitated with NP. It also precipitated PB1 to a smaller extent. We could not determine the status of PB2 or PA because of their poor expression levels. Nevertheless, these results suggested that CNOT4 might display enhanced viral RNA replication after interacting with NP.

### CNOT4 enhances viral NP ubiquitination, whereas the deubiquitinase USP11 reduces it.

**FIG 3:**
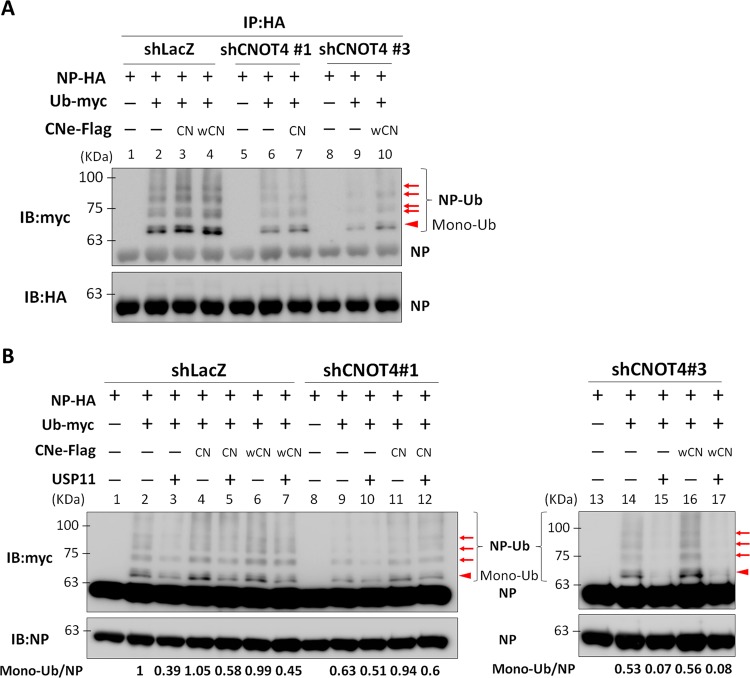
CNOT4 enhances viral NP ubiquitination, whereas the deubiquitinase USP11 reduces it. (A) Knockdown/overexpression of CNOT4 correlates with viral NP ubiquitination. CNOT4 knockdown (shCNOT4 clones) or control shLacZ 293T cells were transfected with Ub-myc and NP-HA, with or without wild-type (CN) or wobble mutant (wCN) CNOT4e-Flag. Cells were subjected to immunoblotting (IB) using HA-agarose. The immunoprecipitated proteins were visualized with anti-HA and anti-myc antibodies. The solid arrowhead indicates the monoubiquitinated NP. The arrows indicate the protein bands cut out for mass spectrometry analysis (see Fig. 5). (B) CNOT4 and USP11 had opposing effects on viral NP ubiquitination. CNOT4 knockdown or control shLacZ 293T cells were cotransfected with NP-HA, Ub-myc, CNOT4e-Flag, and USP11, as indicated, and immunoblotted with HA-agarose. The immunoprecipitated proteins were visualized with anti-NP and anti-myc antibodies. The relative amounts of monoubiquitinated NP (arrow) in each sample are indicated below the gel.

Since CNOT4 has E3 ubiquitin ligase activity (30), we next studied whether CNOT4 triggers ubiquitination of viral RNP, thereby enhancing their function in viral RNA replication. We performed an *in vitro* ubiquitination assay using myc-tagged ubiquitin mixed with cell lysates of the CNOT4 knockdown or control shLacZ 293T cells that had been cotransfected with HA-tagged NP with or without Flag-tagged CNOT4. The lysates were immunoprecipitated with anti-HA antibody–agarose and blotted with anti-HA and anti-myc antibody, respectively. As shown in Fig. 3A, a major band corresponding to the monoubiquitinated species of NP was detected with the anti-myc antibody; this band was significantly weaker in CNOT4 knockdown cells (compare lanes 2, 6, and 9). In contrast, overexpression of CNOT4 or its wobble mutant enhanced NP ubiquitination in both CNOT4 knockdown and control cells (lanes 3, 4, 7, and 10), indicating that the level of ubiquitination of NP was enhanced by CNOT4. However, this monoubiquitinated NP could not be detected by the anti-HA antibody used, probably because of the small amount of this band. Nevertheless, the nature of monoubiquitinated NP was confirmed by mass spectrometry analysis (see below). In addition, there are multiple slower-migrating species that can be detected by anti-myc antibody. PB1 was also ubiquitinated in this ubiquitination assay, but knockdown of CNOT4 did not affect PB1 ubiquitination (see Fig. S1A, lanes 5 and 6, in the supplemental material); therefore, this protein was not further studied.

10.1128/mBio.00597-17.1 FIG S1  PB1 was ubiquitinated, but ubiquitination was not regulated by CNOT4. (A) CNOT4 knockdown or control shLacZ 293T cells were transfected with Ub-myc or PB1-HA, together or not with CNOT4e-Flag. After 48 h posttransfection, cells were lysed and subjected to an immunoprecipitation (IP) assay with HA-agarose. Immunoprecipitated proteins were visualized by Western blotting with anti-HA and anti-myc antibodies. Vertical lines and the arrow indicate the ubiquitinated proteins. Download FIG S1, TIF file, 0.3 MB. Copyright © 2017 Lin et al. 2017 Lin et al. This content is distributed under the terms of the Creative Commons Attribution 4.0 International license.

Previously, we have shown that viral NP can be specifically deubiquitinated by ubiquitin-specific protease USP11, and deubiquitination of NP reduces the efficiency of viral RNA replication (21). We reasoned that CNOT4 and USP11 together could coregulate viral RNA replication through NP ubiquitination and deubiquitination. To test this possibility, CNOT4 knockdown and control shLacZ 293T cells were cotransfected with plasmids that express NP-HA, Ub-myc, CNOT4e-Flag, or USP11. Cell lysates were then subjected to immunoprecipitation and immunoblotting with anti-myc or anti-NP antibodies. The results showed that the NP ubiquitination level was reduced when USP11 was overexpressed (Fig. 3B, lanes 3, 10, and 15) but increased when wild-type (CN) or wobble mutant CNOT4 (wCN) was overexpressed (lanes 4, 6, 11, and 16). Moreover, when coexpressed with both CNOT4 and USP11, the ubiquitination level of NP was again reduced (lanes 5, 7, 12, and 17). These results together indicate that these two enzymes have opposing effects on the ubiquitination level of NP.

### Viral NP is ubiquitinated by CNOT4 *in vitro*, and its ubiquitination does not lead to NP degradation.

**FIG 4:**
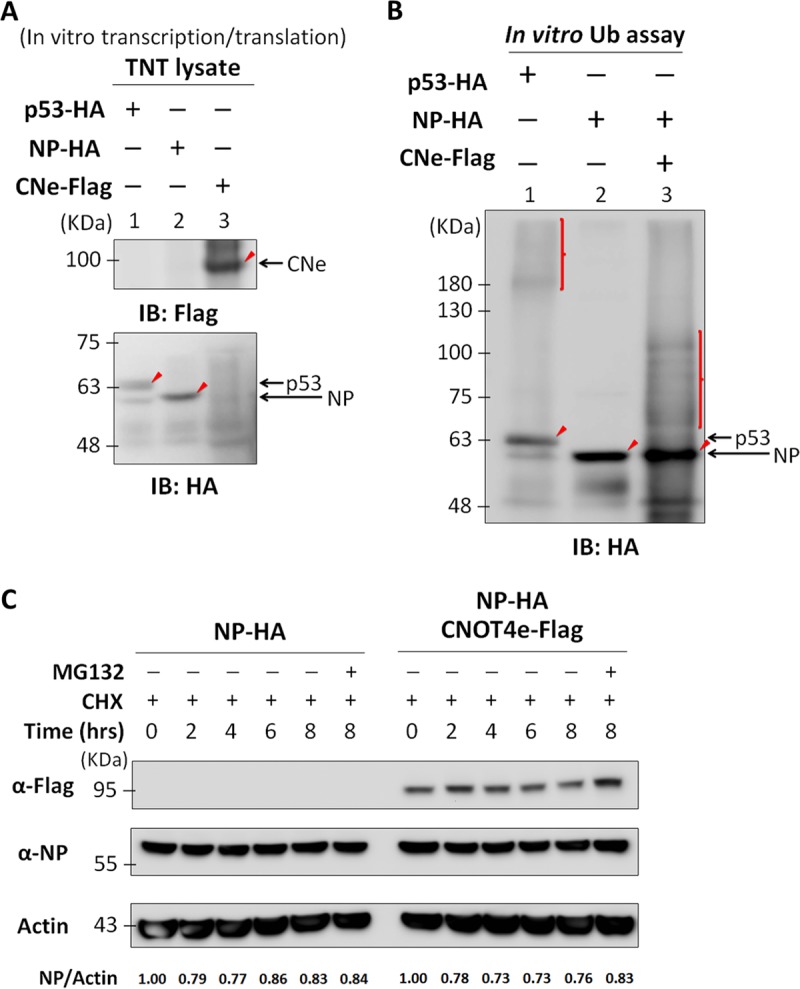
Viral NP is ubiquitinated by CNOT4 *in vitro*, and its ubiquitination does not lead to NP degradation. (A) *In vitro* transcription/translation of NP, CNOT4, and p53. Plasmids carrying NP-HA, CNOT4e-Flag, or p53-HA open reading frames were incubated with TNT lysates at 30°C for 1 h to synthesize the target proteins. The samples were examined by Western immunoblotting (IB) using anti-HA antibody (for p53 and NP) or anti-Flag antibody (for CNe). The respective protein products are indicated by arrows. (B) *In vitro* ubiquitination assay results for NP with CNOT4 E3 ligase. *In vitro*-translated NP-HA and p53-HA TNT lysates were obtained as described for panel A and incubated using a ubiquitination kit with or without *in vitro*-translated CNOT4-Flag at 37°C for 1 h. The ubiquitinated proteins were then detected via immunoblotting using anti-HA antibody. Arrowheads indicate the main protein products without ubiquitination. Vertical lines indicate the ubiquitinated proteins. (C) NP protein stability assay. 293T cells were transfected with plasmids expressing NP-HA, with or without CNOT4e-Flag. At 24 h posttransfection, cells were treated with cycloheximide (50 μM) and MG132 (10 μM) for the indicated lengths of time before lysis. The relative amounts of NP in each sample are indicated below the gel.

To further establish that CNOT4 is a ubiquitin ligase for NP, we used an *in vitro*-synthesized CNOT4 for the ubiquitination assay. First, we used an *in vitro* transcription/translation system, with TNT lysates to synthesize NP-HA and CNOT4e-Flag constructs from the respective plasmids. p53-HA, which is a target of Mdm2 ubiquitin ligase, was included as a control. The proteins synthesized were detected by an anti-HA antibody (detecting NP and p53) and anti-Flag antibody (detecting CNe), respectively. The results showed that all three proteins were synthesized from the *in vitro* transcription/translation-coupled system and had the correct sizes (Fig. 4A). These lysates were then incubated with the autoubiquitination reaction mixtures with or without CNOT4. The reaction products were detected by Western blotting using anti-HA antibody (Fig. 4B). First, the control showed that p53 could be ubiquitinated, since the lysate contained the endogenous Mdm2 E3 ligase for p53 (31) (Fig, 4B, lane 1). By comparison, NP was ubiquitinated only when CNe-Flag was present (compare lanes 2 and 3). This result indicated that CNOT4 is an E3 ubiquitin ligase of NP. Similar to the ubiquitination assay with transfected cell lysates, the ubiquitinated bands made from the *in vitro* reaction mixture were heterogeneous in terms of migration rate and equivalent to proteins in the range of 60 to 100 kDa.

Protein polyubiquitination is known to result in protein degradation through the proteasome (32). Also, a recent publication showed that NP could be polyubiquitinated (15). We therefore examined whether CNOT4 could affect the stability of NP. 293T cells were cotransfected with NP-HA with/without CNOT4e-Flag for 24 h, and then cells were treated with cycloheximide for the indicated lengths of time. The immunoblotting results showed that the amount of NP did not differ whether or not CNOT4 was present (Fig. 4C). The addition of MG132 did not change the protein stability either. We concluded that ubiquitination of NP by CNOT4 does not trigger NP degradation by proteasomes. This result is consistent with our conclusion that CNOT4 is a positive regulator for viral NP.

### Mass spectrometry analysis of NP identified multiple ubiquitination sites, among which the lysine residues located in the RNA-binding domain were most critical for viral RNA transcription/replication.

**FIG 5:**
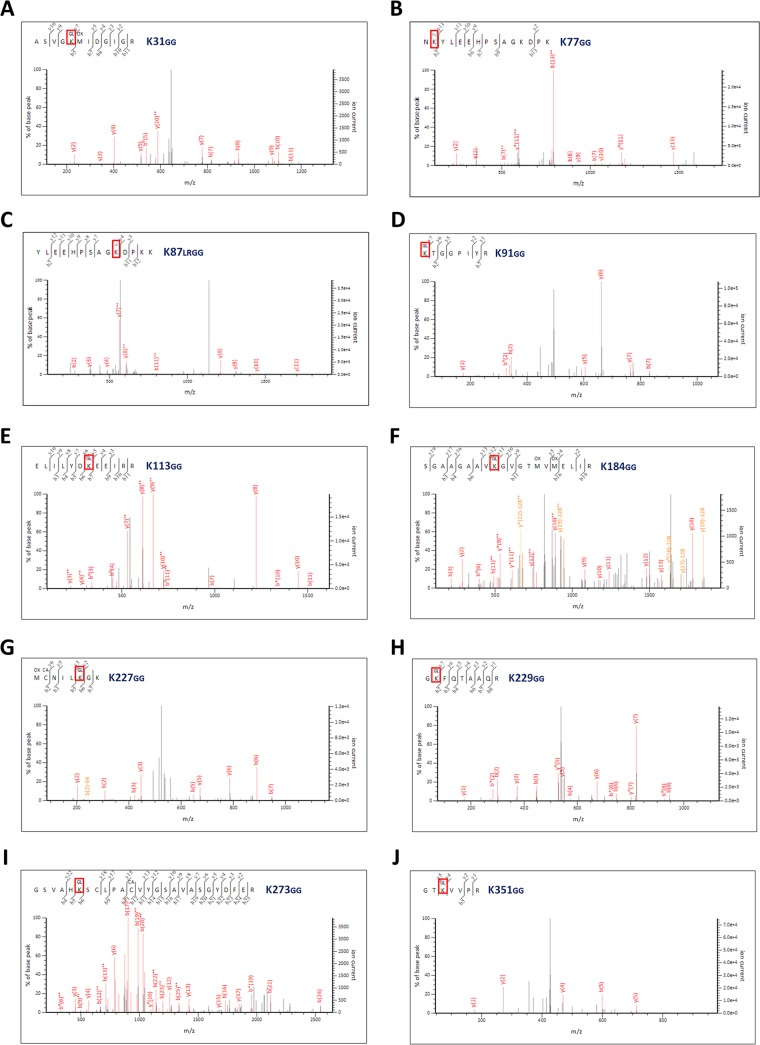
MS/MS analysis of ubiquitinated NP revealed multiple ubiquitination sites. Tandem mass spectra of peptides derived from ubiquitinated NP show ubiquitin conjugation at different residues of ubiquitin with di-Gly modification. Ubiquitinated NP was separated as described for Fig. 3 and isolated using anti-HA–agarose from 293T cells transfected with NP-HA or Ub-myc, with or without CNOT4e-Flag. Protein samples were cut from the indicated protein bands (as for Fig. 3) and further purified (see Materials and Methods). Samples were analyzed by MS analysis. The Mascot search engine was used, with the criteria for searching ubiquitinated lysines marked as LeuArgGlyGly (K_LRGG_) or GlyGly (K_GG_). GG or LRGG modification was observed at the K31 (A), K77 (B), K87 (C), K91 (D), K113 (E), K184 (F), K227 (G), K229 (H), K273 (I), and K351 (J) residues.

Since CNOT4-treated NP showed multiple ubiquitinated bands (Fig. 3), it is possible that there are multiple ubiquitination sites. Alternatively, some NP may be polyubiquitinated. To distinguish these possibilities, we performed mass spectrometry (MS) analysis of NP purified from 293T cells that had been cotransfected with NP-HA or Ub-myc, together (or not) with CNOT4-Flag (Fig. 3). This analysis revealed the identity of the ubiquitin peptides, with a GlyGly or LeuArgGlyGly modification on the lysine residues of the analyzed protein (22). Strikingly, we found that among the 19 lysine residues on NP of the WSN strain of IAV, 10 of them were ubiquitinated (Fig. 5). Previously, we identified K184 as a monoubiquitination site of NP (21); MS analysis of NP confirmed this finding. In addition, it had been reported that five of the lysine residues on NP are conserved across various IAV strains (29). We found that four of them (K113, K184, K229, and K273) are ubiquitinated (21) (Fig. S2A). All of these modifications involve monoubiquitination; no polyubiquitinated NP was detected. These results indicated that NP can be monoubiquitinated at multiple lysine residues.

10.1128/mBio.00597-17.2 FIG S2  Structure and functional domains of NP. (A) The various functional domains of NP and the complete amino acid sequence of NP of IAV strain WSN are shown. The NP regions for binding RNA (black), NP itself (orange), or PB2 (green), and the unconventional nuclear localization signals (NLSs), the bipartite NLS, the cytoplasmic accumulation signal (CAS), and the NP tail loop are indicated. All 19 lysine (K) residues on NP are marked in red; five of them are conserved, based on a recently published report (29), and they are marked in blue. (B) Crystal structure of the influenza A virus NP, modified from that of Ye et al. (33). The five critical lysine residues, including K91, K184, K227, K273, and K351, are shown in red. All of them are marked by an asterisk in panel A. (C) The structure of an influenza virus helical RNP. The RNP complex showing the polymerase (green and brown), two opposite-polarity NP strands (pink and cyanogen), and the terminal NP loop (yellow) (modified from that described by Arranz et al. [34]). The zoomed-in view shows the viral RNA (red and blue) with NP in the helical structure of a viral RNP complex and the relative positions of the lysine residues, shown in yellow and labeled. Download FIG S2, TIF file, 2.4 MB. Copyright © 2017 Lin et al. 2017 Lin et al. This content is distributed under the terms of the Creative Commons Attribution 4.0 International license.

**FIG 6:**
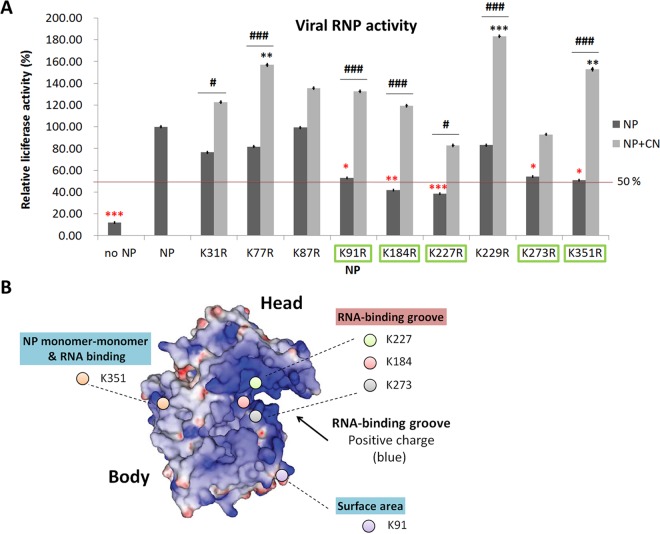
Viral RNP activity of the lysine-to-arginine mutated NP in relationship to its structure; the illustration shows the importance of the lysine residues in the RNA-binding groove of NP. (A) RdRp activities of the various lysine-to-arginine mutants in the absence or presence of exogenous CNOT4. A minireplicon assay for different lysine mutants of NP was performed as described for Fig. 2. Luciferase activity was determined at 24 h posttransfection and normalized to that of the control, WT NP. NP levels (black columns) indicate basal RdRp activity in the absence of exogenous CNOT4; NP plus CN levels (gray columns) indicate RdRp activity in CNOT4-overexpressing cells. Red asterisks indicate the KR mutants that had statistically significantly lower RdRP activities than those of the wild-type NP; they are further highlighted with green boxes. Black asterisks indicate the KR mutants that had statistically significantly higher RdRP activities than the wild-type NP in the presence of overexpressed CNOT4. The black # sign indicates the mutants whose RdRP activities were enhanced by CNOT4 to a statistically significant level (means ± SD, *n* = 3). *, *P* < 0.05; **, *P* < 0.01; ***, *P* < 0.001 (versus WT NP). #, *P* < 0.05; ###, *P* < 0.001 (versus KR-NP), based on a one-way ANOVA with Tukey’s multiple-comparison test. (B) Locations of the critical lysine residues on the NP molecule. The three-dimensional structure of the influenza A virus NP was modified from that described by Chenavas et al. (44). K184, K227, and K273 are located in the RNA-binding groove of NP (with blue indicating a positive charge); K91 and K351 are located on the NP surface.

To address the functional significance of ubiquitination of each individual lysine, we individually mutated each of the potentially ubiquitinated lysine residues to arginine. Except for K113, mutation of which had previously been shown not to affect RdRp activity (21), we assayed RdRp activities of all the remaining NP mutants via the minireplicon method and compared the activites with that for the wild-type NP. Five of the mutants (K91, K184, K227, K273, and K351) were found to have statistically significantly lower RdRp activity than the wild-type NP by approximately 50% or more (Fig. 6A). Further, overexpression of CNOT4 enhanced RdRp activity of several of the mutants by almost 100% or more (statistically significant). Some mutations did not affect RdRp activity, but overexpressed CNOT4 induced higher RdRp activity than seen with wild-type NP (statistically significant). Thus, CNOT4 enhanced RdRp activity of all NP mutants, probably because multiple lysine residues contributed to the CNOT4-mediated functions. Among all the lysine residues, K91, K184, K227, K273, and K351 (Fig. 6A) are considered most critical for the viral RdRp activities induced by CNOT4-mediated ubiquitination.

To understand the possible mechanism of enhancements of RdRp activity by ubiquitination of these five lysine residues, we further analyzed the locations of these lysines on the NP molecule (Fig. 6B). The crystal structure of NP, modified from that reported by Ye et al. (33), showed that all five lysine residues are located on the surface of NP (Fig. S2B). They can further be divided into two groups: those in contact with the RNA and those not in contact. In the structural model of the viral helical RNP complex, modified from that reported by Arranz et al. (34), three of the lysines (K184, K227, and K273) are located on the RNA-binding groove of NP (Fig. S2C). The others (K91 and K351) are located on the NP surface. K351 also binds viral RNA at a site near the monomer-monomer-binding region, where NP forms a helical RNP structure (Fig. S2C). Together, these lysine residues are on the exposed area of NP; thus, ubiquitination on these residues is not expected to disrupt the basic structure of NP, but it may alter protein-RNA or protein-protein interactions. Mutations changing lysine to arginine also would not be expected to change the positive charge of the amino acid residue through interfering with its binding with the negatively charged viral RNA. Moreover, our data showed that none of the nine single lysine-to-arginine mutations completely lost the ability to respond to CNOT4 (Fig. 6A), suggesting that CNOT4 can target multiple lysines on the NP protein. Taken together, we conclude that viral NP has multiple monoubiquitination sites, and ubiquitination of the lysine residues on the RNA-binding groove and other RNA-binding regions are most critical for high efficiency of viral RNA transcription/replication.

## DISCUSSION

Our studies found that NP is modified by ubiquitination, which is mediated by a ubiquitin ligase, CNOT4. The presence of ubiquitin is crucial for the function of NP in viral RNA transcription/replication. NP is the most abundant viral protein in infected cells. It exists as a component of the viral ribonucleoprotein structure, complexed with viral RNA and viral polymerase subunits in the virion and in the cells to participate in viral RNA transcription/replication. Thus, NP protein interacts with itself (35, 36) and with RNA (37), viral RdRp (PA, PB1, and PB2) (38), and cellular factors (39–43). Previously, it was demonstrated that phosphorylation of NP at Ser165 regulated the oligomerization status and RNA-binding activity of NP (44). In the monomeric structure, this serine is situated at the interface between the two lobes of the core of NP, close to the C terminus. Moreover, our earlier studies showed that NP is modified at K184. Thus, protein modifications are important parameters in the structure and functions of the viral RNP complex.

Surprisingly, K184 is not the only lysine residue that is ubiquitinated. Mass spectroscopic analysis showed that as many as 10 lysine residues on the NP could be ubiquitinated by CNOT4. Most of these residues are on the surface of the NP. Thus, the posttranslational ubiquitination of NP is not expected to change the basic structure of the NP. Rather, these modifications will likely change the interaction of NP with the homologous or heterologous molecules or with viral RNA. Indeed, three potential ubiquitinated lysine residues (K184, K227, and K273) are located on the RNA-binding groove of NP (33). It is conceivable that ubiquitination of these residues enhances the RNA-binding activity of NP, resulting in an increase of viral RdRp activity. This finding is reminiscent of our previous report’s indicating that a cellular factor, RRPB1, can increase RNA-binding activity (45) and enhance RdRp activity.

Another class of lysine residues is those situated outside the RNA-binding groove of NP, as exemplified by K91 and K351. Based on the influenza A virus helical RNP structure and the NP crystal structure (34, 46), it is believed that when NP forms a polymer strand, K351 is located on NP monomer-monomer-binding site (Fig. S2C). The viral RNA can also bind with K351 in the helical RNP crystal structure. Ubiquitination of K351 may stabilize NP strand conformation, or it may increase the NP-RNA binding ability. It is reassuring that all these ubiquitinated lysines are located at the surface of the NP molecule. Thus, ubiquitination of these residues will not change the basic structure of the NP molecule, but rather they will change its interactions with other molecules.

Curiously, it has been reported that NP is polyubiquitinated, which causes degradation of NP through the proteasome pathway (15). Thus, it was proposed that ubiquitination negatively regulates the functions of NP by degrading NP. However, in our MS analysis, we did not detect any polyubiquitination of NP. We did detect multiple heterogeneous ubiquitinated species in the *in vitro* ubiquitination reactions with CNOT4 (Fig. 4). However, the MS-MS analysis showed that all of these bands consisted of monoubiquitinated species. There was no apparent correlation between the electrophoretic mobility of the bands and the nature of the ubiquitinated species. It is likely that these higher-molecular-weight species represent ubiquitinated proteins complexed with cellular protein or NP protein with multimonoubiquitination. It is not clear whether ubiquitination can take place at more than one lysine residue on the same NP molecule or whether there is a limit on the number of ubiquitination sites per NP molecule. The variation in the amounts of these multiubiquitination bands may reflect the variation in the physiological conditions of viral growth or the experimental procedures. Our previous (21) and current work showed that NP function can be positively and negatively regulated through ubiquitination and deubiquitination by CNOT4 and USP11, respectively. This mechanism provides a more precise way of regulating NP function. Nevertheless, polyubiquitination of NP may occur under some conditions and potentially is a general mechanism for regulation of the NP function.

Influenza A virus undergoes rapid evolution. We have aligned the RNA sequence of viral NP from the years 1918 to 2016 and obtained consensus sequences for H1N1 and H3N2 NP. There are 13 lysine residues that are conserved among H1N1, H3N2, and WSN NP proteins, including K91, K184, K227, and K273. (K351 has mutated to arginine in some virus strains, suggesting that it may be less critical for the structure or function of NP.) The evolution of viral proteins in adaptation to the host’s physiological systems is important for a virus to survive in nature. Favorable features will remain and be kept in the species through evolution. The diversity and complementarity of multiple monoubiquitination sites of NP may affect the efficiency of viral RNA replication. Thus, these conserved lysine residues may play certain roles in the IAV life cycle.

CNOT4, a subunit of the Ccr4-Not transcription complex, is evolutionarily conserved and important for regulation of mRNA synthesis and decay (47). The E3 ligase activity of the human CNOT4 subunit, orthologous with yeast Not4p, implies that the Ccr4-Not complex is involved in protein ubiquitination/degradation pathways. Our results show that CNOT4 regulates NP ubiquitination and enhances viral RNA replication. It is possible that the interplay between CNOT4 and the Ccr4-Not complex is linked to the modification of NP to regulate viral RNA synthesis. Thus, it is possible that the host Ccr4-Not transcription complex may play some roles in viral replication during viral infection.

## MATERIALS AND METHODS

### Cell culture.

The human lung carcinoma cell line A549 was maintained in F-12K medium (Corning Cellgro) supplemented with 10% fetal bovine serum (Thermo Scientific) and antibiotics (100 U/ml penicillin G and 100 g/ml streptomycin). Madin-Darby canine kidney (MDCK) and HEK293T cells were maintained in Dulbecco’s modified Eagle’s medium (Corning Cellgro) supplemented with 10% fetal bovine serum and antibiotics (100 U/ml penicillin G and 100 g/ml streptomycin). All cells were cultured at 37°C and in 5% CO_2_.

### Virus and plasmids.

The A/WSN/33 (WSN) strain of influenza A virus was used in this study. All of the plasmids required for lentivirus production were provided by the National RNAi Core Facility, Academia Sinica, Taiwan. The five pLKO.1-shRNA vectors used for knockdown of CNOT4 were TRCN0000015213 (shCNOT4-1), TRCN0000015215 (shCNOT4-2), TRCN0000015216 (shCNOT4-3), TRCN0000234728 (shCNOT4-4), and TRCN0000234729 (shCNOT4-5). The pLKO.1-shLacZ control plasmid was TRCN0000072240 (shLacZ). The wobble mutants of CNOT4, used for the rescue experiments, contained the same amino acid sequence of the CNOT4 protein but different coding RNA sequence. The resultant construct was verified by sequencing forward to correspond to isoform e of CNOT4. These plasmids were named pFLAG-CNOT4e and pFLAG-wobble CNOT4e (wobble CNOT4 isoform e for shCNOT4-3; ACAGAGCCTCTTCACATCAGAAACAATCCC to ACAGAGCCT***T***TTCAC***G***TC***G***GAAAC***G***ATCCC; the changed nucleotides are shown in bold italics), respectively.

### Antibodies and reagents.

Anti-HA and anti-myc antibodies were purchased from Santa Cruz Biotechnology (sc-805 and sc-789). Anti-IAV NP antibody was purchased from Abcam, Inc. (catalog number AB20343). Anti-Flag (DYKDDDDK tag) antibody was purchased from Pierce (MA1-91878). MG132, an S26 proteasome inhibitor, and cycloheximide were purchased from Sigma-Aldrich (catalog numbers C2211 and C6255). All Alexa Fluor-conjugated secondary antibodies used for immunofluorescence were procured from Molecular Probes (Invitrogen), and DAPI came from Sigma-Aldrich. M-PER (mammalian protein extraction reagent), anti-HA affinity matrix, and anti-Flag affinity matrix were obtained from Thermo Scientific.

### Secondary screen.

A549 cells were seeded at a density of 8,000 cells per well in a 96-well assay plate and transduced with RNAi lentiviruses; shRNA-positive cells were selected using puromycin (3 μg/ml). At 5 days posttransduction, the cells were infected with strain A/WSN/33 (WSN) for 6 h and harvested for immunofluorescence staining using anti-NP antibody. The images were captured using the ImageXpress Micro XLS wide-field high-content screening system (Molecular Devices).

### Immunofluorescence staining.

A549 cells infected with influenza A virus were washed with phosphate-buffered saline (PBS), then fixed with 4% paraformaldehyde and permeabilized with PBS containing 0.2% Triton X-100, followed by blocking with 0.25% bovine serum albumin (BSA) and probing with anti-NP antibody (1:800) overnight at 4°C. After extensive washes, cells were incubated with anti-mouse Alex Fluor-conjugated antibody (1:400) for 1 h, and cell nuclei were labeled with DAPI.

### Lentivirus production.

The TransIT-LT1 transfection reagent (MirusBio) was used for lentiviral production in 293T cells with a packaging construct (pCMVΔR8.91), an envelope construct (pMD.G), and different pLKO.1-shRNA vectors according to the protocol on the RNAi Core website. At 16 h posttransfection, the medium was changed and replaced with BSA-containing medium. At 40 h posttransfection, virus was harvested and replaced with BSA-containing medium. The medium was harvested again at 64 h posttransfection, and then the cells were discarded. The virus was pooled and spun at 1,250 rpm for 5 min to pellet the cells that were collected during harvesting. Virus was stored at −80°C.

### RNAi knockdown.

All RNAi reagents were obtained from the National RNAi Core Facility. The effective lentivirus-based RNAis for CNOT4 used in this study were shCNOT4-1: 5′-CCCTGTAGTTTCTGGACGTTT-3′ (3′-UTR region), shCNOT4-2, 5′-GCCTCTTCACATCAGAAACAA-3′ (CDS region), shCNOT4-3, 5′-GCCCTTGGAGATAGATGATAT-3′ (CDS region), shCNOT4-4, 5′-CAGTATAGGGAACGGTGATAA-3′ (CDS region), shCNOT4-5, and 5′-ACAGAGTCACAGTCGTTATTC-3′ (CDS region). The control RNAi was shLacZ, 5′-TCGTATTACAACGTCGTGACT-3′. Cells were seeded on 6-well tissue culture plates (2 ml per well) and incubated overnight. Cells were maintained in culture medium containing 8 mg/ml Polybrene. The RNAi lentivirus was added to cells at a multiplicity of infection (MOI) of 3 and incubated overnight, and then selected with 3 mg/ml puromycin for 3 days. The CNOT4 stable knockdown or control cell lines, derived from A549 or 293T cells, were cultured in medium containing puromycin and used in the study.

### Western blot analysis.

Cells were lysed by using M-PER (Thermo Scientific) with additional protease inhibitors. Samples were separated by SDS-PAGE and transferred onto a PolyScreen polyvinylidene difluoride membrane (PerkinElmer). The membrane was immunoblotted with the indicated primary and appropriate secondary antibodies and detected using WesternBright ECL spray (Advansta). The images were captured using a BioSpectrum imaging system (UVP, Inc., LCC).

### Quantitative RT-PCR.

Total cellular RNA was extracted using a High Pure RNA isolation kit (Roche Diagnostics) according to the manufacturer’s protocol. cDNA was synthesized using the SuperScript III first-strand synthesis system (Invitrogen). The primers for reverse transcription were oligo(dT)_20_ and IAV-specific RT primer (uni-12, 5′-AGCAAAAGCAGG-3′). Real-time PCR analysis followed the standard TaqMan method with the Universal Probe Library (UPL) system and LightCycler 480 apparatus (Roche Diagnostics). Glyceraldehyde-3-phosphate dehydrogenase (GAPDH) was used as a control for normalization of cellular RNA and intracellular viral RNA levels. The sequences of primers and probes were as follows: for the IAV NP segment, sense 5′-GATGGAGACTGATGGAGAACG-3′, and antisense, 5′-TCATTTTTCCGACAGATGCTC-3′ with universal probe 59; for CNOT4, sense, 5′-CGGTGGTTTCTTGTGAGGAC-3′, and antisense, 5′-AGCTAAAATGTAGGACTTTGACGAC-3′, with universal probe 21; for GAPDH, sense, 5′-AGCCACATCGCTCAGACAC-3′, and antisense, 5′-GCCCAATACGACCAAATCC-3′ with universal probe 60.

### PFU assay.

To determine PFU, viral supernatants were collected and PFU were determined in a plaque assay. Monolayers of MDCK cells were cultured in 6-cm dishes and incubated with serial dilutions (10× each) of 1-ml aliquots of viral supernatants at 37°C. After 1 h, 3 ml of 0.5% *α*-minimal essential medium containing agarose was then added to the cells, left at room temperature until it set, and then incubated at 37°C. At 48 h postinfection, the agarose layer was removed and the plaques were visualized with 0.1% crystal violet solution.

### Coimmunoprecipitation assay.

293T cells were transfected in 6-well dishes by use of X-tremeGENE transfection reagents (Roche Diagnostics) according to the manufacturer’s directions. Approximately 48 h after transfection, cells were washed with PBS and lysed in 400 μl of M-PER (Thermo Scientific) with additional protease inhibitors. After centrifugation at 4°C for 15 min, 20 μl of cell extract was stored as input sample; the remaining protein was incubated with 15 μl anti-HA or anti-Flag–agarose (Thermo Scientific) and rotated at 4°C for 4 h. Samples were washed three times with Tris-buffered saline (TBS) plus 0.05% Tween 20 (TBS-T), and then 25 μl nonreducing sample buffer (2×) was added and the mixture was heated for 5 min. Samples were analyzed by Western blotting using the indicated antibodies.

### *In vitro* ubiquitination assay (cell transfection system).

HEK293T cells were transiently transfected with mammalian expression plasmids for Myc-tagged Ub or HA-tagged NP, with or without Flag-tagged CNOT4. After transfection for 48 h, cells were harvested using M-PER (Thermo Scientific) and centrifuged to remove the insoluble fraction. The clarified supernatant was subjected to immunoprecipitation with anti-HA–agarose (Thermo Scientific). The ubiquitinated NP was detected by Western blotting using anti-myc or anti-HA antibody.

### Minireplicon assay.

HEK293T cells were cotransfected with plasmids for expression of viral proteins PB1, PB2, PA, NP (IAV polymerase complex), and pPolI-Luc (expressing negative-sense RNA containing a luciferase reporter gene to serve as a substrate for viral RdRp) as described in reference 21. *Renilla* luciferase activity was used as an internal control to normalize transfection efficiency results. At 24 h posttransfection, cells were collected and the luciferase activity was measured using Dual-Glo luciferase (Promega) according to the manufacturer’s protocol.

### MS/MS analysis of ubiquitinated NP.

Ubiquitinated NP was isolated by using anti-HA beads and cells transfected with NP-HA or Ub-myc, together (or not) with CNOT4e-Flag. Prior to tandem mass spectrometry (MS/MS) analysis, the bound proteins were separated by polyacrylamide gel electrophoresis and stained with Coomassie blue; the ubiquitinated species were cut and then washed with buffer containing 25 mM NH_4_HCO_3_ and 40% methanol. Samples were incubated with buffer containing 10 mM dithiothreitol (DTT) in 100 mM NH_4_HCO_3_ for 1 h at 37°C for reduction and alkylation. Eluted proteins were digested with enzyme solution containing 12.5 ng/μl of trypsin in 25 mM NH_4_HCO_3_–10% acetonitrile and incubated overnight at 37°C. After trypsinization, cells were treated with trifluoroacetic acid (TFA) and clarified by centrifugation for extraction of peptides. Samples were desalted on a C_18_ ZipTip pipette tip (Millipore) and lyophilized for MS analysis.

Shotgun proteomic identifications via Nano LC-nano ESi MS/MS analysis were performed on a NanoAcquity system (Waters, Milford, MA) connected to an LTQ-Orbitrap XL hybrid mass spectrometer (Thermo Fisher Scientific, Bremen, Germany) equipped with a nanospray interface (Proxeon, Odense, Denmark). Peptide mixtures were loaded onto a 75-μm inner diameter, 25-cm length C_18_ bridged ethylene hybrid column (Waters, Milford, MA) packed with 1.7-μm particles with a pore width of 130 Å and were separated using a segmented gradient in 60 min from 5% to 40% solvent B (acetonitrile with 0.1% formic acid) at a flow rate of 300 nl/min and a column temperature of 35°C. Solvent A was 0.1% formic acid in water. The mass spectrometer was operated in the data-dependent mode. Briefly, survey full-scan MS spectra were acquired in the Orbitrap (*m/z* 350 to 1,600) with the resolution set to 60,000 at *m/z* 400 and automatic gain control (AGC) target at 106. The 10 most intense ions were sequentially isolated for collision-induced dissociation MS/MS fragmentation and detection in a linear ion trap (AGC target at 7,000) with previously selected ions dynamically excluded for 90 s. Ions with singly or unrecognized charge state were also excluded. All the measurements in the Orbitrap were performed with the lock mass option for internal calibration.

The MS and MS/MS raw data were processed by using Raw2MSM v.1.10 and searched against the customized Swiss-Prot *Homo sapiens* database and in-house-generated WSN NP protein sequences using the Mascot search engine (v.2.4.0; Matrix Science, Inc., Boston, MA) through Proteome Discoverer (v. 1.4.0.288; Thermo Scientific). Search criteria used were trypsin digestion, variable modifications set as carbamidomethyl (C), oxidation (M), ubiquitinated LeuArgGlyGly (K), and GlyGly (K) allowing up to two missed cleavages, and mass accuracy of 10 ppm for the parent ion and 0.6 Da for the fragment ions. Two target values for a decoy database search were applied: a strict false discovery rate (FDR) of 0.01 and a relaxed FDR of 0.05. Ubiquitination sites and peptide sequence assignments contained in MASCOT search results were validated by manual confirmation from the raw MS/MS data.

### *In vitro* ubiquitination assay (*in vitro*-translated protein system).

The protein analysis system used *in vitro*-translated protein for ubiquitination studies. Plasmids of NP-HA, CNOT4e-Flag, and p53-HA were used in the TNT Quick Coupled Transcription/Translation systems (Promega) first to translate the desired ubiquitin ligases and their potential substrates. Each reaction mixture contained 1 μg of DNA and 30 μl of TNT master mix, and mixtures were incubated at 30°C for 1 h. The translated protein products were detected by Western blotting using anti-HA and anti-Flag antibodies.

The *in vitro* ubiquitination assays were performed according to the auto-ubiquitinylation kit (Enzo Life Sciences) instructions, using *in vitro*-translated CNOT4 or p53 according to the manufacturer’s instruction. Each reaction mixture (50 μl, final volume) contained 2.5 μl 20× E1, 2.5 μl 20× E2 (UbcH5b), 5 μl 10× Ub E3 ligase buffer, 5 μl 10× ubiquitin, 1 μl 50 mM DTT, 2.5 μl Mg-ATP, 15 μl CNOT4e-Flag TNT lysate, or 2.5 μl 20× E3 control (Mdm2) (6 μM) and 15 μl of NP-HA or p53-HA lysates as described above. p53 served as a positive control, since it can be ubiquitinated by the endogenous Mdm2 ubiquitin ligase. The reaction mixtures were incubated at 37°C for 1 h. The ubiquitinated protein species were detected by Western blotting using anti-HA antibody.

### Statistical analysis.

All the data are reported as the sample means ± the standard deviations (SD). Multiple comparisons were performed using a univariate analysis of variance (ANOVA). All the data were processed by using PRISM version 5.03. For Fig. 1B, C, and E and 2A and B, the data were analyzed by one-way ANOVA with Dunnett’s multiple-comparison test, with 95% confidence intervals for the difference. For Fig. 2C and 6A, the data were analyzed by one-way ANOVA with Tukey’s multiple-comparison test, with 95% confidence intervals for the difference.
