# Supplementary figures and images for: CNOT4-Mediated Ubiquitination of Influenza A Virus Nucleoprotein Promotes Viral RNA Replication
Source: mBio. 2017 May 23;8(3):e00597-17. doi: 10.1128/mBio.00597-17 (PMC5442456; doi:10.1128/mBio.00597-17)

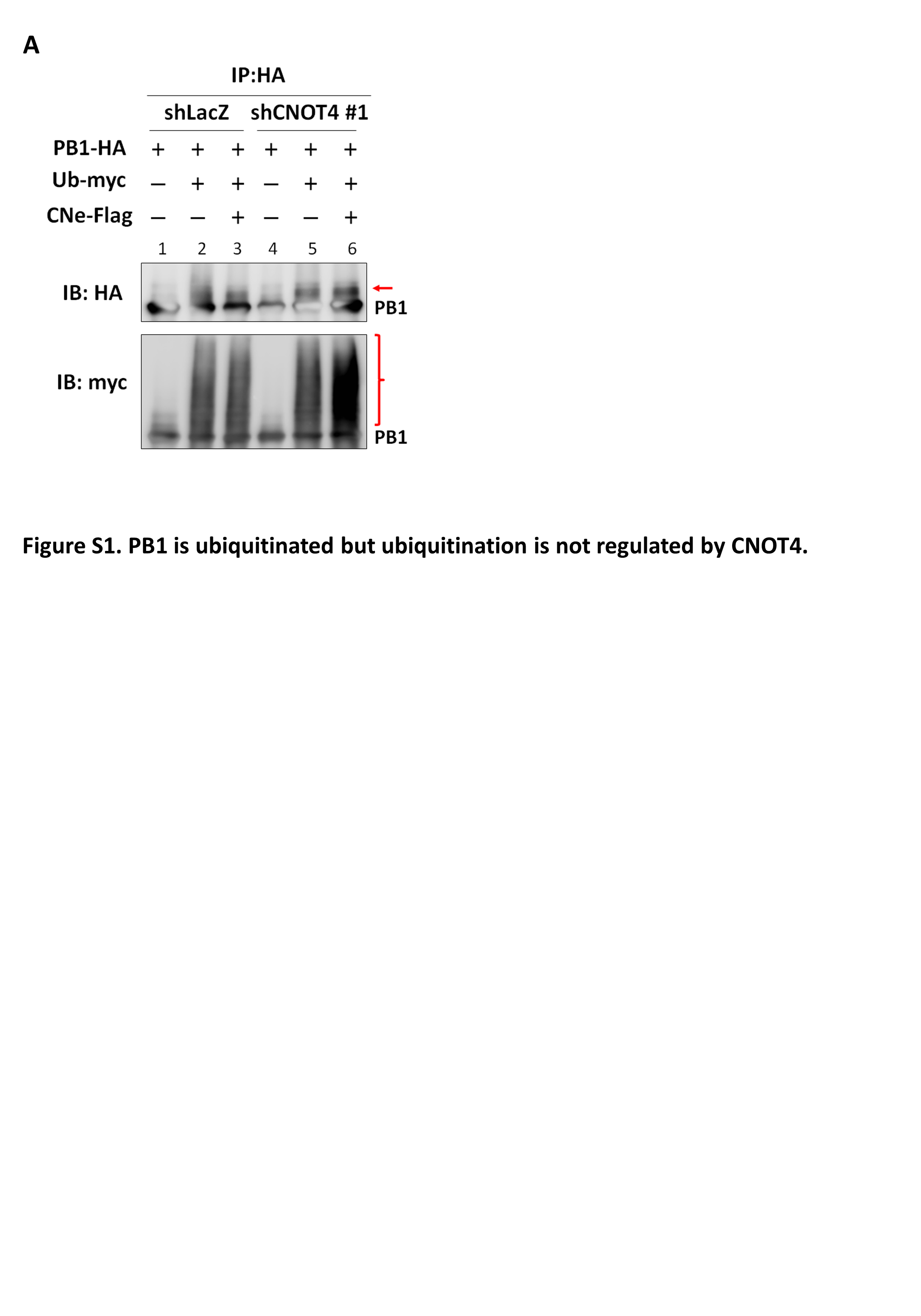

Supplement: FIG S1 [file mbo003173318sf1.tif]

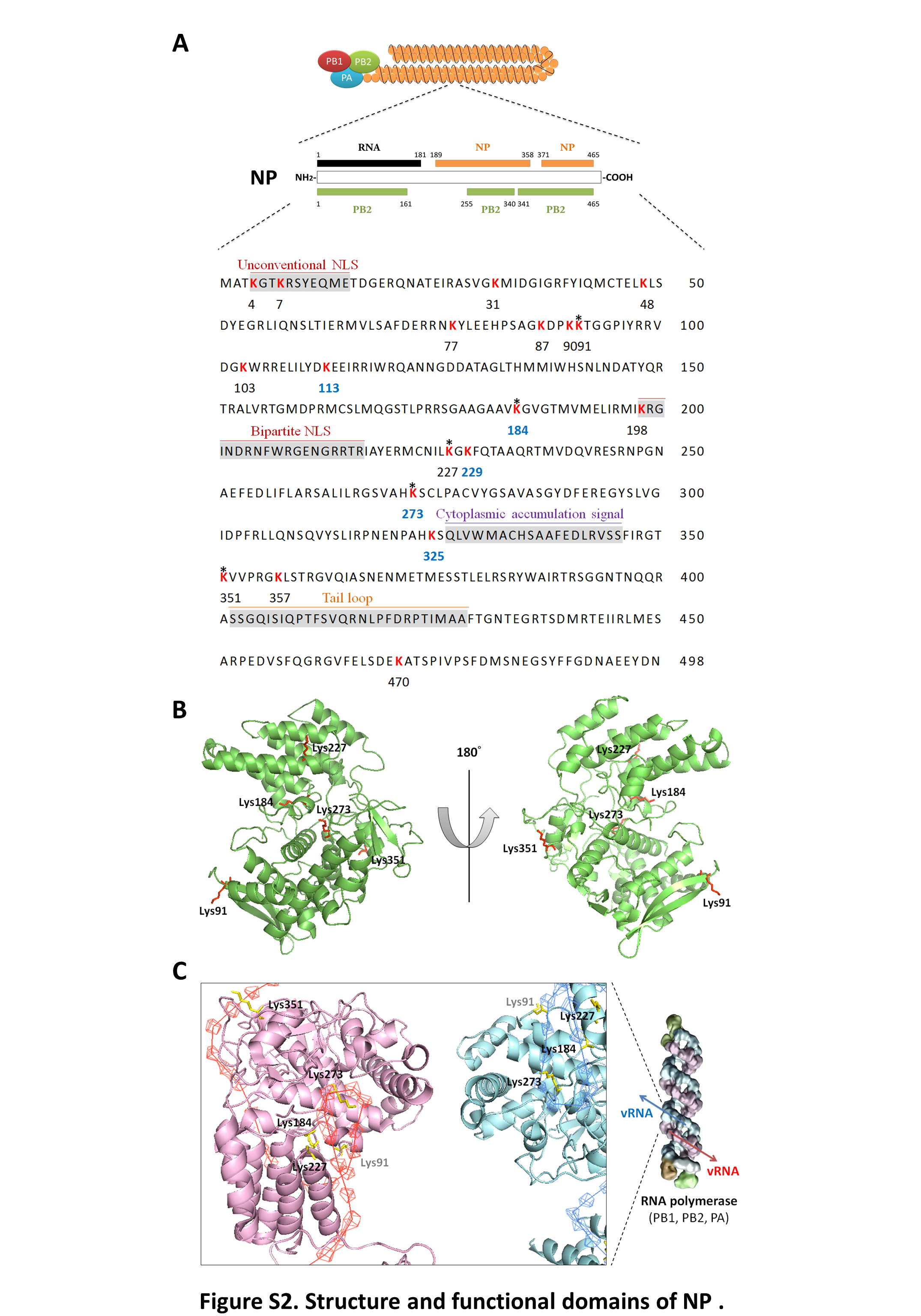

Supplement: FIG S2 [file mbo003173318sf2.tif]
